# First-Time Mothers' Enjoyment of Breastfeeding Correlates with Duration of Breastfeeding, Sense of Coherence, and Parental Couple and Child Relation: A Longitudinal Swedish Cohort Study

**DOI:** 10.1155/2020/8194389

**Published:** 2020-06-19

**Authors:** Agnes Granberg, Anette Ekström-Bergström, Caroline Bäckström

**Affiliations:** ^1^Psykologiteamet, Göteborg, Sweden; ^2^Department of Health Sciences, University West, Trollhättan, Sweden; ^3^School of Health Sciences, University of Skövde, Post Box 408, S 541 28, Skövde, Sweden

## Abstract

**Objectives:**

Many women do not reach their own breastfeeding goals regarding duration of breastfeeding. Different factors influence breastfeeding, and to learn more about breastfeeding within a multidimensional and longitudinal perspective, further research is needed. Therefore, the aim of the present study was to investigate diverse factors correlated with first-time mothers' enjoyment of breastfeeding and breastfeeding duration, between childbirth and two years after birth.

**Methods:**

In a prospective longitudinal cohort study, 324 newly become mothers were followed. The Spearman correlation test was used to investigate factors correlated with the degree to which mothers enjoy breastfeeding and the duration of breastfeeding. The Mann–Whitney test was conducted for comparisons of demographic characteristics between mothers who did or did not breastfeed.

**Results:**

Among the mothers, 99.2% initiated breastfeeding after birth. Frequencies of breastfeeding were 54.8% at six months, 9.1% at one year, and 1.0% at two years. The degree to which the mother enjoyed breastfeeding was correlated positively with (1) the duration of breastfeeding, (2) more positive feelings for and relation to the child, (3) the partner's perceived relation to the child, (4) a higher sense of coherence, and (5) stronger perceived parental couple's relationship. Longer breastfeeding duration was correlated positively with (1) a higher degree of enjoyment of breastfeeding, (2) more positive relation to the child, and (3) stronger perceived parental couple's relationship. Additionally, breastfeeding during the first two hours after birth, more positive feelings for and relation to the child, and a higher degree of enjoyment of breastfeeding were more frequently reported among breastfeeding mothers, in comparison with not breastfeeding mothers.

**Conclusion:**

Mothers' subjective experience from breastfeeding, sense of coherence, and couple relationship with partner and relationship with the child are valuable factors in regard to breastfeeding.

## 1. Introduction

Various studies show that many women are unable to reach their own breastfeeding goals regarding duration of breastfeeding [1–3]. In an American study, 60% of mothers had a shorter breastfeeding duration than they had wanted [4]. Breastfeeding is recognised as promoting various health benefits for mother and child, and the World Health Organization (WHO) recommends exclusive breastfeeding for the first six months of life and at least some breastfeeding during an infant's first years [5, 6]. Breastfeeding duration in line with WHO's recommendations, however, is uncommon in developed countries [7, 8]. One explanation to this could be that mothers from higher income families have better financial conditions to procure breast milk substitutes [8].

In 2016, almost 95% of infants born in Sweden were breastfed at the age of one week [9]. As in other developed countries around the world, breastfeeding rates in Sweden drop considerably until the age of six months, with exclusive breastfeeding dropping from 74% at one week to 14% at six months and 50% of children receiving any breastfeeding at six months [9].

The percentage of mothers breastfeeding one year after birth varies internationally with, for example, 35% in Norway, 27% in the USA, 16% in Sweden, and 1% in the United Kingdom [8]. Most women in developed countries have ended their breastfeeding when the child reaches the age of one year. A review of the literature studying breastfeeding beyond one year of age in western countries revealed that women who breastfeed after one year of age and therefore do not follow the norms of breastfeeding can experience judgement, negative comments, stigma, and the need to conceal their breastfeeding [7].

When initiating breastfeeding, many women experience some type of barrier to success, such as the perception of insufficient milk supply and difficulties to latch [10]. Research from recent qualitative studies in Sweden connects breastfeeding difficulties to a feeling of lostness as a mother, feelings of loneliness, and the need to deal with shattered expectations and thoughts, such as being of no use to the infant. The studies highlight the importance of seeing breastfeeding not merely as a way to feed the child, but also as an existential challenge for the mother and as a way towards closeness between mother and child [11, 12].

While breastfeeding is one of the challenges confronting new parents [10], research has also shown that breastfeeding is capable of reducing a mother's physiological and experienced stress, facilitating positive affect, and having a positive effect on maternal sensitivity and care [13].

An individual's ability to cope with the stressors encountered in life has previously been explained through the person's sense of coherence (SOC) [14]. Previously, it has been described that high SOC can help parental couples conceptualise the world as meaningful and manageable, including how parents perceive and cope with the challenges that come with childbirth and parenthood [15–17]. Also, mothers with higher SOC are more likely to breastfeed for a longer period [18]. While previously a person's life in which no radical life events had occurred was considered stable [19], later studies have described SOC as a continuously changing process throughout a person's life [20], where life events such as childbirth and the first years of parenthood can change SOC within both a positive and negative direction [21, 22]. A study following a group of parents from pregnancy until six months of age showed an increase in both quality of the couple's relationship (QDR36) and SOC, during the first week after childbirth. Thereafter, the measures for the QDR36 and SOC decreased significantly when the child had reached six months of age [23]. However, which factors influence mothers' breastfeeding may vary both individually among the mothers and nationally and internationally. It is clear that breastfeeding is a complex phenomenon and knowledge is lacking about diverse factors influencing mothers' enjoyment of breastfeeding and the breastfeeding duration. To learn more about breastfeeding within a multidimensional and longitudinal perspective, further research is needed. Therefore, the aim of the present study was to investigate diverse factors correlated with first-time mothers' enjoyment of breastfeeding and breastfeeding duration, between childbirth and two years after birth.

## 2. Methods

The present study is a prospective longitudinal cohort study that is a part of a larger ongoing study: the *Study of Parental Support,* a study that follows first-time mothers and their partners from pregnancy until eight years after birth. The overall aim with the *Study of Parental Support* is to explore parents' individual resources and experiences from childbirth, as well as effects of professional and social support. The larger research study consists of both qualitative research with interviews and written narratives and quantitative research with follow-up questionnaires. Some research has been previously published within the *Study of Parental Support* [23–27]. Specifically, the quantitative research with follow-up questionnaires from the first week until two years after birth is considered for the present study.

### 2.1. Setting and Participants

The present study was conducted in a county in southwestern Sweden, with approximately 280,000 inhabitants. The county includes urban as well as suburban and rural areas and is in that way representative of the general Swedish population. In the area, there is one hospital with a labour ward and a postnatal unit with approximately 2700 births per year. The participants in the present study were mothers recruited by midwives at the county's postnatal unit during the first week after birth. The recruitment took place between June 2014 and April 2016. The inclusion criteria for the study were Swedish-speaking, first-time mothers who gave birth to a singleton infant. No further exclusion criteria were used.

### 2.2. Data Collection

Data for the present study were collected at four different points in time (Q): first week after birth (Q1), six months after birth (Q2), one year after birth (Q3), and two years after birth (Q4). The questionnaires included several different measurements. From the questionnaires used in the larger research study, the “*Study of parental support*,” only those that were in line with the aim of the present study were investigated.

**Figure 1:**
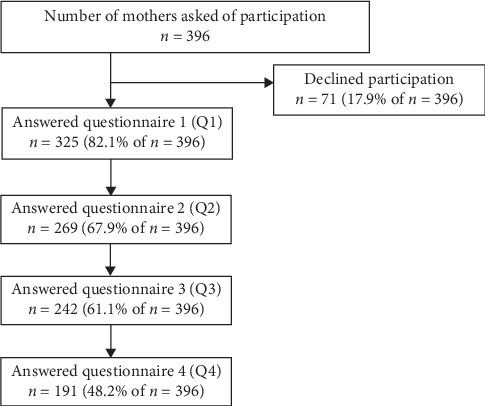
Flowchart of longitudinal study, with response rate presented in *n* and (%). Q1 = first week after childbirth, Q2 = six months after childbirth, Q3 = one year after childbirth, and Q4 = two years after childbirth.

The participants filled out web-based questionnaires created in the computer system *Education Survey Automation Suite (EvaSys)*. At Q1, the participants could answer the questionnaire at the postnatal unit. For those participants who did not complete Q1 at the postnatal unit, they received it via email during the first week after birth. The following questionnaires (Q2–Q4) were sent via email. Up to three reminders for each time point were sent to the participants that had not yet filled out the questionnaires. The number of participants who accepted to take part in the study and the response rate for each questionnaire are presented in Figure 1.

### 2.3. Pilot Studies

Two pilot studies were conducted before the present study to explore parents' experiences from responding to the questionnaires. Within the first pilot study, 16 parents filled out the questionnaires in paper form. Within the second pilot study, 22 parents filled out the questionnaires in the web-based form, which the present study later used. Some of the participants from each study (five in total) described their experiences of responding to the questionnaires. The results of the pilot studies showed that the information given to the participants and composition of the questionnaires were generally understandable and manageable. Before data collection for the present study started, minor changes were made within the questionnaires to incorporate participant information. The participants included within the pilot studies were not included in the present study.

### 2.4. Questionnaires

#### 2.4.1. Sociodemographical Factors

Questions concerning the following sociodemographical factors were included in the questionnaires at different time points throughout the study: age of the participant, years of education attained before first child's birth, years of couple relation with partner, and perceived economy (four-graded scale: 1 = I perceive my financial situation as strained; 4 = I perceive my financial situation as very good).

#### 2.4.2. Duration of Breastfeeding

Questions concerning if the mothers were breastfeeding or not were included in the questionnaires throughout the study (Q1–Q4). The mothers were asked regarding exclusive or partial breastfeeding. Within the current study, exclusive breastfeeding is defined as mothers exclusively giving their child breast milk as the only nutrition. Mothers who responded that they gave their child breast milk in combination with formula and/or solid foods (such as porridge and/or “normal food”) were defined as partially breastfeeding their children. Breastfeeding in this study refers to any breastfeeding if not otherwise specified. In Q1, the mothers were asked whether or not they breastfed their child during the first two hours after birth. Included within Q4 was also a question concerning whether or not the mothers were willing to breastfeed again, in case they would have another child.

#### 2.4.3. Mother to Infant Relations and Feelings Scale (MIRF-Scale) and Enjoying Breastfeeding

To assess the mother's relation to and feelings for her child, the MIRF-scale was used [28–30]. The MIRF-scale is a validated [28, 30] seven-graded Likert scale (ranging from 1 to 7) consisting of two different parts: (1) the first part assesses the mother's perceived relation to her child (7 items) and (2) the second part assesses the mother's perceived feelings for her child (7 items). Within the first part, the mother's perceived relationship to her child is questioned, followed by different statements such as “I know what my baby wants/I do not know what my baby wants.” Originally, the index is calculated by summarising the seven statements. Within the present study, however, the index of the first part of the MIRF-scale is analysed both with and without the item concerning breastfeeding: “I enjoy breastfeeding/I do not enjoy breastfeeding,” which means that the score index for the mother's perceived relation to her child (the first part of the MIRF-scale) is based on six (score range 7–42) *or* seven (score range 7–49) items within the present study. The higher the score, the stronger the mother's perceived relation to child. Within the present study, the variable that describes the index score of the first part of the MIRF-scale is named “*Mother's perceived relation to the child (“Enjoyment of breastfeeding included”* or “*Enjoyment of breastfeeding not included”).*”

In the present study, the item “I enjoy breastfeeding/I do not enjoy breastfeeding” is also analysed separately and used as a variable named “*Enjoyment of breastfeeding*” (score range 1–7); the higher the score, the more did the mother perceive herself enjoying breastfeeding.

Within the second part of the MIRF-scale, the mother's feelings for her child are assessed with a question concerning the mother's perceived contact with her child. This question is followed by seven items constructed of opposing word pairs, such as “secure/insecure.” The items consist of a seven-point response scale ranging from 1 to 7. The index score (score range 7–49) summarises the mother's feelings for her child. The higher the score, the stronger the mother's perceived feelings for the child. Within the present study, the variable that describes the index score of the second part of the MIRF-scale is named “*Mother's perceived feelings for the child*.”

To assess how the mother perceived the partner's feelings for the child, the same questions (items) given to the mother about her contact with the child (the second part of the MIRF-scale) were also given to the mother regarding how she perceived her partner's contact with their child. Those seven items were developed for the present study and tested within the two pilot studies. The index score (score range 7–49) summarises how the mother perceived the partner's feelings for their child. The higher the score, the stronger the partner's perceived feelings for the child. The variable that describes the index score is named “*Mother's partner's perceived feelings for the child*.” The MIRF-scale and the questions regarding the “Mother's partner's perceived feelings for the child” are included within Q1–Q4.

#### 2.4.4. Sense of Coherence (SOC-13)

The mother's SOC was assessed with the instrument named SOC-13. It consists of 13 items divided into three dimensions: *Comprehensibility* (e.g., “Do you have very mixed-up feelings and ideas?”), *Manageability* (e.g., “How often do you have feelings that you are not sure you can control”), and *Meaningfulness* (e.g. “Until now your life has had no clear goals–very clear goals and purpose”). Each item is scored with a Likert scale, ranging from 1 to 7 [14, 31]. The Swedish version has been used for several years [32], and a validation study on SOC-13 used on pregnant women has been carried out earlier [33]. SOC-13 is assessed with an index for the whole scale (score range 13–91); the higher the score, the higher the SOC. SOC-13 was included within Q1–Q4.

#### 2.4.5. Quality of the Couple's Relationship (QDR36)

The mothers' perceived quality of the couple's relationship was assessed with the scale *Quality of Dyadic Relationship* (QDR36) [34–36]. QDR36 has been tested and validated regarding its psychometric properties [36]. It consists of 36 items divided into five dimensions: *Dyadic Consensus* (e.g., “How often do you and your partner agree or disagree handling family finances”), *Dyadic Cohesion* (e.g., “How often do you think you and your partner have a stimulating exchange of ideas”), *Dyadic Satisfaction* (e.g., “How often do you and your partner quarrel?”), *Dyadic Sensuality* (e.g., “How often do you hug your partner now?”), and *Dyadic Sexuality* (e.g., “How often do you think your partner pays attention to your sexual needs?”). Each item is scored with a Likert scale, ranging from 1 to 6. The perceived quality of the couple's relationship is assessed with an index for the whole scale, created by the sum of the mean values from the separate dimensions [36] (score range 5–30); the higher the score, the higher the perceived quality of couple relationship. QDR36 is included within Q1–Q4.

### 2.5. Statistical Analysis

The Statistical Package for the Social Sciences (SPSS), version 25, was used for the statistical analyses of the data. Inconsistent and possibly incorrect answers were controlled for and eliminated for the statistical analysis. Descriptive statistics were carried out in order to present the sociodemographics and other measures for the participants (Table 1). Index and dimensions for measurements included were calculated. Cronbach's alpha was calculated to evaluate internal consistency for *SOC-13*, *QDR36*, *MIRF-scale part one and two*, and *Mother's partner's perceived feelings for the child* (Table 2).

To consider the distribution of the variables, histograms and scatter plots were made. These showed that the following variables were not normally distributed: “*Enjoyment of breastfeeding*” (at Q1-Q2); *“Duration of breastfeeding”*; *SOC-13* (at Q3 and Q4); “*Mother's perceived relation to the child”* (at Q1–Q4); and “*Mother's perceived feelings for the child”* (at Q1–Q4). Since neither of the two variables “*Enjoyment of breastfeeding*” or “*Duration of breastfeeding*” was normally distributed, the nonparametric Spearman's correlation test was used to analyse correlated factors with (1) the degree to which mothers enjoy breastfeeding at six months and one year of age and (2) the duration of breastfeeding from childbirth until two years of age.

The Mann–Whitney test was conducted for comparison between first-time mothers who did or did not breastfeed: (1) during the first two hours after birth (Q1), (2) at six months after birth (Q2), and (3) at one year after birth (Q3) (Table 3). Analyses for comparison were carried out for the mothers' *Age*; *Education; Perceived economy*; *Years of parental couple relationship*; *MIRF-scale part one and two*; *Mother's partner's perceived feelings for the child*; *SOC-13*; and *QDR36.*

Using the nonparametric Friedman's test, comparisons were made between Q1 and Q2; Q2 and Q3; Q3 and Q4; Q1 and Q3; Q1 and Q4; Q2 and Q4, in relation to the variable “*Enjoyment of breastfeeding*.” These analyses were carried out to explore changes over time in regard to first-time mothers' enjoyment of breastfeeding. After a statistically significant Friedman's test, the Wilcoxon signed rank test for post hoc testing was performed. The results of the Wilcoxon signed rank test are presented within the results section.

*p* values < 0.05 were considered significant and *p* values <0.1 were interpreted as tendencies. For this study, the STROBE guidelines for cohort studies have been used (see “Supplementary material (available here)”).

### 2.6. Nonrespondents

To investigate differences between respondents who filled in both the first (Q1) and the last (Q4) questionnaire and nonrespondents who did not fill in both Q1 and Q4, the Mann–Whitney test was used for ordinal variables (*Age*, *Marital status*, *Perceived economy*, *SOC-13*, *QDR36*, *MIRF-scale part one and two*, and *Mother's partner's perceived feelings for the child*) and chi-square test for discrete variables (*Skin-to-skin contact after birth* and *Any breastfeeding*).

### 2.7. Ethical Statement

This study was approved by the Regional Ethical Review Board in Gothenburg (Dnr 197–14; Dnr T 623–14). All participants received information both verbally and in writing about study rationale, the anticipated benefits and potential risks of the study, the right to refuse to participate, and the right to withdraw at any time. The identities of the participants were kept confidential, and in the reporting of the data, none of the participants could be identified.

## 3. Results

### 3.1. Participants

In total, 325 first-time mothers accepted to participate in the present study. The number of participants eligible for each analysis is presented in Figure 1 and Table 1. The characteristics of the participants are presented in Table 1. When comparing participants who filled in both Q1 and Q4 with participants who did not fill in both Q1 and Q4, there were no significant differences in relation to their age, marital status, perceived economy, SOC-13, perceived quality of parental couple relationship (QDR36), mother to infant relations and feelings (MIRF-scale), and skin-to-skin contact after birth nor to their breastfeeding at Q1.

### 3.2. Breastfeeding Frequencies between First Week and Two Years after Birth

Among the mothers, 99.2% responded that they were breastfeeding at Q1 and 54.8% were breastfeeding at Q2. The frequencies of breastfeeding mothers were 9.1% at one year after birth (Q3) and 1.0% at two years after birth (Q4). Frequencies of exclusive and partial breastfeeding are presented in Table 1.

At Q4, 80.1% of the mothers answered that they were willing to breastfeed again if they would have a second child; 5.2% of the mothers responded that they were not willing to breastfeed again; and 8.4% of the mothers did not know whether they would be willing to breastfeed again or not. At Q4, 20.9% of the mothers responded that they had given birth to a second child and 22.5% of the mothers responded that they had been breastfeeding a second child, which indicates that more mothers answered the breastfeeding question in comparison with the amount of mothers who answered the question regarding if they had given birth to a second child (Table 1).

### 3.3. Enjoying Breastfeeding

The mothers rated how much they enjoyed breastfeeding on a seven-graded Likert scale: “Enjoyment of breastfeeding,” within the MIRF-scale. The mothers reported the highest values in relation to “Enjoyment of breastfeeding” at Q3 (*M* = 6.1) and the lowest values at Q1 (*M* = 5.6), in comparison with the other points of time (Q2: *M* = 5.9; Q4: *M* = 6.0) (Table 2). Results from the Wilcoxon signed rank test showed that the mothers reported significantly higher values in relation to “Enjoyment of breastfeeding” at Q4 compared to Q1 (*p* < 0.000) and Q2 (*p*=0.008). No other significant results were shown from the Wilcoxon signed rank test regarding “Enjoyment of breastfeeding” at different points in time. Results from the Wilcoxon signed rank test are not presented in tables.

#### 3.3.1. Factors Correlated with Mothers' Enjoyment of Breastfeeding and the Duration of Breastfeeding

Results from Spearman's correlation analysis showed that the mothers' rated enjoyment of breastfeeding at Q2 was correlated positively with (1) higher rated enjoyment of breastfeeding at Q3 (*r*_*s*_ *=* 0.529, *p*=0.006); (2) a longer breastfeeding duration (*r*_*s*_ = 0.241, *p*=0.005); (3) a higher SOC-13 at Q1 (*r*_*s*_ = .263, *p* < 0.000) and Q2 (*r*_*s*_ = 0.233, *p*=0.007); and (4) a stronger perceived parental couple's relationship (QDR36) at Q1 (*r*_*s*_ = 0.240, *p*=0.002). Likewise, mothers' relation to the child (MIRF-scale part one) was correlated positively with the mothers' rated “*Enjoyment of breastfeeding*” at Q2 (item “*Enjoyment of breastfeeding*” included at Q3: *r*_*s*_ = 0.473, *p*=0.002; Q4: *r*_*s*_ = 0.505, *p*=0.012; item “*Enjoyment of breastfeeding*” not included at Q2: *r*_*s*_ = 0.314, *p* < 0.000; Q3: *r*_*s*_ = 0.252, *p*=0.005; Q4: *r*_*s*_ = .215, *p*=0.022). In addition, the mothers' higher rated enjoyment of breastfeeding at Q2 correlated positively with mothers' higher rated perceived feelings for the child (MIRF-scale part two) (Q2: *r*_*s*_ = 0.359, *p* ≤ 0.000; Q3: *r*_*s*_ = 0.257, *p*=0.004; and Q4: *r*_*s*_ = 0.190, *p*=0.046) and mothers' higher rated partner's perceived feelings for the child (Q2: *r*_*s*_ = 0.235, *p*=0.005) (Table 4).

At Q3, the mothers' higher rated enjoyment of breastfeeding was correlated positively with mothers' higher rated (1) enjoyment of breastfeeding at Q2 (*r*_*s*_ = 0.529, *p*=0.006); (2) perceived relation to the child (MIRF-scale part one, item “*Enjoyment of breastfeeding*” included) at Q3 (*r*_*s*_ = 0.589, *p*=0.002); and (3) perceived relation to the child (MIRF-scale part one, item “*Enjoyment of breastfeeding*” not included) at Q2 (*r*_*s*_ = 0.445, *p*=0.026) and Q3 (*r*_*s*_ = 0.424, *p*=0.035) (Table 4).

A longer breastfeeding duration was correlated positively with mothers' higher reported (1) enjoyment of breastfeeding at Q2 (*r*_*s*_ = 0.241, *p*=0.005) and (2) perceived relation to the child (MIRF-scale part one, item “*Enjoyment of breastfeeding*” included) at Q4 (*r*_*s*_ = 0.527, *p* ≤ 0.000). Significant and nonsignificant results from Spearman's correlation analysis are presented in Table 4.

#### 3.3.2. Comparisons between Breastfeeding and Not Breastfeeding from Birth until Two Years of Age

There were significantly more mothers who breastfed during the child's first period awake after birth who also breastfed at one week after birth (Q1), compared to those mothers who did not breastfeed during the first hours after birth (*z* = −3.31, *p*=0.001) (Table 3).

A larger amount of the mothers who were breastfeeding at Q2 reported higher scores for “Mother's perceived relation to the child” (*z* = −2.49; *p*=0.013) in comparison with mothers who did not breastfeed at that point in time. Furthermore, a larger amount of the mothers who breastfed at Q3 did report higher scores for (1) “Mother's perceived relation to the child” (*z* = −2.10; *p*=0.036) and (2) “Mother's perceived feelings for the child” at six months (Q2) (*z* = −2.18; *p*=0.030) (Table 3).

A larger amount of the mothers who breastfed at Q1 were willing to breastfeed a possible following child, compared with mothers who did not breastfeed at Q1 (*z* = −4.75; *p*=0.000). Likewise, a larger amount of the breastfeeding mothers at Q2 responded that they were willing to breastfeed a possible following child (*z* = −4.72; *p*=0.000). More mothers who breastfed the child at Q3 were willing to breastfeed a possible following child (*z* = −2.07; *p*=0.038) and more of them had given birth to another child at Q4 (*z* = −2.06; *p*=0.040) (Table 3).

When comparing breastfeeding mothers with nonbreastfeeding mothers in relation to their SOC-13 and QDR36, no significant differences were found at any point in time (Table 3). Significant and nonsignificant results from the Mann–Whitney tests are presented in Table 3.

## 4. Discussion

For the present study, the aim was to investigate diverse factors correlated with first-time mothers' enjoyment of breastfeeding and breastfeeding duration, between childbirth and two years after birth. The results revealed that mothers' higher reported enjoyment of breastfeeding was correlated positively with a longer breastfeeding duration, higher perceived relation to and feelings for the child, as well as higher SOC, higher quality of parental couple relationship, and higher perceived partner's feelings for the child. A longer breastfeeding duration was also correlated with mothers' higher perceived relation to the child and quality of parental couple relationship. In addition, breastfeeding during the first two hours after birth, more positive feelings for and relation to the child and a higher degree of enjoyment of breastfeeding were more frequently reported among breastfeeding mothers, in comparison with not breastfeeding mothers, at different points in time.

In this study, the results showed that more mothers were breastfeeding at one week after birth (99.2%), in comparison with previous reports on Swedish first- and multipara mothers (95%) [9]. At six months, the amount of mothers breastfeeding was slightly higher (54.8%) in comparison with previous reports (50%) [9]. The results from the present study revealed that a longer duration of breastfeeding correlated positively with the mothers' higher reported enjoyment of breastfeeding, which is in line with earlier research [34]. The majority of mothers breastfeeding at all points of time in the study scored high on the subscale “*Enjoyment of breastfeeding*” in the MIRF-scale. Regardless of the seemingly positive experience of breastfeeding, most of the mothers breastfeeding at six months and at one year after childbirth weaned within the following months. The possible influence of a stigma regarding breastfeeding until and beyond one year [7] might be the explanation for this, which future studies could investigate further.

Earlier research shows that just as breastfeeding is seen as a protective factor against developing postpartum depression [37], early breastfeeding difficulties have been suggested as a risk factor for depression [38] as well as for stress and other forms of psychological distress for the mother [39]. At the same time, maternal psychological distress has also been shown to be a risk factor for breastfeeding difficulties [40, 41]. Results from this study showed that higher rated SOC correlated positively with a higher rated enjoyment of breastfeeding at six months after birth. It might be that a first-time mothers' higher SOC could strengthen her ability to cope with the challenges that come with parenthood and initiating breastfeeding, resulting in an easier path towards being able to enjoy breastfeeding. In addition, previous research has shown that mothers with a higher SOC are more likely to breastfeed for a longer time, compared with mothers with a lower SOC [18]. Taking into consideration these results, a mother's SOC seems to be related to both her breastfeeding duration and her breastfeeding experience. Therefore, healthcare professionals should strive to strengthen the mothers' sense of coherence during the childbearing period, particularly since SOC previously has been shown to decrease during the first months after childbirth [20–22].

The positive correlation at six months after birth between “*Enjoyment of breastfeeding*” and “*Mother's partner's perceived feelings for the child*” could mean that first-time mothers who experience that the other parent has a well-functioning relationship with the child can more easily share both the enjoyment and the challenges that come with the transition to parenthood and can therefore enjoy breastfeeding more. It might also be that a partner who is perceived as having a better relationship to the child takes more responsibility in the family with the child, and therefore, the mother might experience more support from her partner, as shown in an earlier study [34]. Nevertheless, the mother's capacity for being sensitive to her child's needs and signals is vital for the attachment [42]. Professional support from healthcare professionals has been proven to be important in facilitating both a mother's sensitivity to her child's signals as well as for the child's needs and the mother's caring response [43]. Furthermore, professional support is helpful for the mother to adapt to her new role [44] and her well-being [45]. Therefore, health professionals should see breastfeeding not merely as a way to feed the child, but also as an existential challenge for the mother and as a way towards closeness between mother and child [11, 12]. However, further exploration is needed in relation to associations between perceived parent-to-child relations and mothers' enjoyment of breastfeeding.

Interestingly, the present study showed a significant correlation between the first-time mothers' perceived quality of parental couple's relationship (QDR36) and their breastfeeding duration as well as enjoyment of breastfeeding. This highlights the relevance of healthcare professionals supporting the parental couple's in their relationship to each other, especially since it is known that the perceived parental couple's relationship decreases after the birth of the first child [23]. In addition, earlier research has shown that the time during which the partner was present after labour was correlated with the duration of breastfeeding in first-time mothers. Feelings of confidence when the baby was six to twelve months were retrospectively correlated with the mother's feelings of confidence with the partner during childbirth [46].

Among the mothers breastfeeding one year after birth, the degree to which they enjoyed breastfeeding was positively correlated with how they had perceived the relation with their child at six months, in the MIRF-scale. While most studies investigating breastfeeding and the relation between mother and child focus on characteristics of the mother and factors influencing the mother, there is also research suggesting that characteristics of the child, such as the child's temperament, can have an effect on breastfeeding practices [47]. There are also studies showing that how the mother experiences breastfeeding can have a causal effect on the mother's relation to the child. An intervention study from Sweden revealed that professional breastfeeding support can affect the mothers` feelings for their infants and enjoyment of breastfeeding [29] which indicates the need of satisfactory professional support.

The socioeconomic factors (age, education, perceived economy, and length of parental couple relationship) that are commonly reported to be associated with the duration of breastfeeding [48–50] were analysed within this study. Remarkably, in this study, none of the included socioeconomic factors were significantly correlated with neither the duration nor the enjoyment of breastfeeding. Earlier research has highlighted the importance of placing the relationship between educational level and breastfeeding in a social context [51] which could be an explanation of the lack of significant correlations. In a Swedish study examining breastfeeding until six months of age in a sample of 51–415 children, born between 2004 and 2011, the researchers found that the socioeconomic gap between the mothers' duration of breastfeeding had been narrowed, though it still existed. In that study, they hypothesised that the narrowed gap could be explained by the fact that highly educated women, who once started the trend of longer breastfeeding in Sweden during the 1980s and 1990s, now might be the first to follow a new trend of breastfeeding for a shorter time [50]. While the present study has a much smaller sample and therefore might not find small correlations, the results still suggest that it is unlikely that socioeconomic factors can explain much of what affects the mothers' experience of and the duration of breastfeeding in Sweden. When considering these results within an international perspective, it is valuable to note that the mothers included within the present study were living in Sweden which is a country that provides parents with a relatively long period of parental leave. Parents living within other countries may not have the same opportunities for parental leave, and therefore, the breastfeeding in other countries might be more affected by socioeconomic factors.

Result from this study showed that there were significantly more mothers who breastfed during the child's first period awake after birth who also continued to breastfeed at one week after birth. In addition, mothers who were breastfeeding at six months reported higher scores for “Mother's perceived relation to the child,” compared to mothers who did not breastfeed during the first hours after birth. These results are in line with previous findings [29]. Since earlier studies show a relation between the duration of breastfeeding and the mothers' attitude towards breastfeeding [52, 53] and breastfeeding behaviour, such as breastfeeding in public [54], it would be interesting to further investigate the relationship between enjoyment of breastfeeding and the more societal and culturally related attitudes and experienced norms towards breastfeeding.

When studying the descriptive data for some of the measurements, it can be noted that most of the participants had scored the maximum score or close to the maximum. This is true both for the item “*Enjoyment of breastfeeding*” and the three measurements “*Mother's perceived relation to the child*,” “*Mother's perceived feelings for the child*,” and “*Mother's partner's perceived feelings for the child*,” within the MIRF-scale. This was dealt with by using nonparametric tests in the statistical analysis. However, for future studies, it could be relevant to modify these measurements or develop other measures to be able to catch the full spectrum of the variation intended to be studied.

Cronbach's alpha was calculated to evaluate internal consistency, and the results showed high values for several of the included measures *(SOC-13*, *QDR36*, and *MIRF-scale part two)* and lower values for the MIRF-scale part one. For future studies, the MIRF-scale part one is in need of development. This scale would, perhaps, benefit from improvement with a few items such as “*I enjoy spending time with my child*” or “*I feel secure in my relation to my child*.”

Comparing the mothers' answers from different questions and from Q1–Q4, the answers were not always coherent. For example, regarding the duration of breastfeeding and partial and exclusive breastfeeding. One explanation for this could be that later given answers tended to be less trustworthy because it is difficult to remember behaviours many months after, especially those charged with emotion [55] which breastfeeding memories could be. However, the longitudinal design of this study made it possible to discover these patterns and it is recommended that such challenges are considered when designing future studies about breastfeeding. A limitation of a longitudinal design is, though, the reduced number of participants to follow up. For the present study, 48.2% of the mothers responded at Q4, which corresponds to a total loss at 51.8%. However, the results from analyses between respondents who answered at Q4 and nonrespondents who did not answer at Q4 showed no differences which could be considered as a study strength.

## 5. Conclusions

A large majority of the first-time mothers in the study enjoyed breastfeeding and would choose to breastfeed again if they had another child. The degree to which the mother enjoyed breastfeeding was correlated with the duration of breastfeeding, a higher SOC, more positively perceived feelings for and relation between mother and child, and a more positive perception of how the mother perceived her partner's relation to the child. A more positively perceived couple's relation was correlated to both a longer duration of breastfeeding and a higher degree of enjoyment of breastfeeding.

The present study indicates that health personnel who are interested in supporting the first-time mothers' coping ability and a positive relationship between parent and child could benefit from asking more about the subjective experience a mother has breastfeeding her child. Such questions could be, for example, “How do you experience breastfeeding?” “What does breastfeeding mean to you and your family?” and “In what way are you and your wellbeing affected by the breastfeeding?” From learning more about the mother and families' subjective experiences, the support for the family should include a focus on how feelings of meaningfulness, manageability, and enjoyment can be found in the experience of breastfeeding and parenting in general. It is also important for health professionals to take into consideration the quality of the couple's relationship, which can, for example, be strengthened by an environment that enables the family to stay together after birth, providing a unique opportunity for the newly becoming parents to establish bonds with each other and with the child.

## Figures and Tables

**Table 1:** Overview of characteristics at different times (Q) throughout the study.

	First-time mothers (*n*)	*M* (SD)	MD, range	Frequencies in percentage (%)
**Age** (years) at Q1	310	27.9 (4.1)	27, 18–43	
**Country of birth** at Q4	**191**			
Sweden	178			93.2
Nordic country	1			0.5
Europe, other than Nordic country	3			1.6
Non-European country	8			4.2
Missing	1			0.5
**Education** (years) at Q4	184	14.4 (2.0)	15, 9–19	
**Civil status** at Q1	**325**			
Married	72			22.2
Cohabiting	212			65.2
Not living together	4			1.2
Missing	37			11.4
**Length of parental couple relationship** (years) at Q4	146	7.7 (3.4)	7.3–24	
**Still in the same parental couple relationship**				
At Q3	239			98.8
At Q4	185			96.9
**Perceived economy** at Q1	**325**			
Very good	54			16.6
Good	148			45.5
Sufficient	75			23.1
Strained	10			3.1
Missing	38			11.7
**Perceived economy** at Q2	**269**			
Very good	16			5.9
Good	112			41.6
Sufficient	109			40.5
Strained	32			11.9
Missing	0			0.0
**Perceived economy** at Q3	**242**			
Very good	16			6.6
Good	85			35.1
Sufficient	105			43.4
Strained	35			14.5
Missing	1			0.4
**Perceived economy** at Q4	**191**			
Very good	32			16.8
Good	97			50.8
Sufficient	50			26.2
Strained	12			6.3
Missing	0			0.0
**On parental leave**				
At Q2	258			95.9
At Q3	167			69.0
At Q4	50			26.2
**Weeks of any breastfeeding**	**248**	27.5 (19.0)	27.0, 0–105	
First week after birth (at Q1)	246			99.2
4 weeks after birth	223			89.9
11 weeks after birth	186			75.0
21 weeks after birth	149			60.0
26 weeks after birth (at Q2)	136			54.8
36 weeks after birth	74			29.8
42 weeks after birth	50			20.2
57 weeks after birth	10			4.0
67 weeks after birth	5			2.0
91 weeks after birth	2			1.0
**Breastfeeding first week after birth**				
Exclusive breastfeeding	218			67.1
Partial breastfeeding	50			15.4
**Breastfeeding six months after birth**				
Exclusive breastfeeding	35			13.0
Partial breastfeeding	136			50.1
**Breastfeeding one year after birth**				
Partial breastfeeding	22			9.1
**Breastfeeding two years after birth**				
Partial breastfeeding	2			1.0
**Pregnant**				
At Q3	20			8.3
At Q4	52			27.2
**Given birth to a second child**				
At Q3	2			0.8
At Q4	40			20.9
**Breastfed a second child** at Q4	**191**			
Yes, one child	41			21.5
Yes, two or more children	2			1.0
**Willingness to breastfeed a second child** at Q4	**191**			
Yes	153			80.1
No	10			5.2
Do not know	16			8.4
**Missing**	**12**			6.3

*Questionnaires*: Q1: first week after birth; Q2: six months after birth; Q3: one year after birth; Q4: two years after birth. *Values*: *n* = number of participants; *M* = mean; SD = standard deviation; MD = median.

**Table 2:** Overview of index, dimensions, and outcome measures at different times throughout the study.

Measurement	Q1: first week after birth					Q2: six months after birth					Q3: one year after birth					Q4: two years after birth				
	*n*	*M* (SD)	MD	Range	*α*	*n*	*M* (SD)	MD	Range	*α*	*n*	*M* (SD)	MD	Range	*α*	*n*	*M* (SD)	MD	Range	*α*
Enjoyment of breastfeeding	278	5.6 (1.4)	6.0	1.0–7.0		148	5.9 (1.2)	6.0	1.0–7.0		26	6.1 (0.9)	6.0	4.0–7.0		2.0	6.0 (0)	6.0	6.0–6.0	
MIRF-scale part one, *item “Enjoyment of breastfeeding” included*	271	42.4 (4.4)	43.0	25.0–49.0	0.62	187	40.6 (3.2)	41.0	24.0–49.0	0.54	65	44.3 (4.4)	45.0	29.0–49.0	0.57	47	42.2 (3.8)	43.0	32.0–48.0	0.33
MIRF-scale part one, *item “Enjoyment of breastfeeding” not included*	284	36.8 (3.6)	37.0	23.0–42.0	0.56	265	38.3 (3.4)	39.0	20.0–42.0	0.52	240	38.7 (3.2)	39.0	24.0–42.0	0.69	191	38.8 (3.0)	39.0	25.0–42.0	0.44
MIRF-scale part two	277	44.7 (4.8)	46.0	19.0–49.0	0.90	263	47.4 (3.0)	49.0	32.0–49.0	0.91	240	47.7 (2.7)	49.0	29.0–49.0	0.91	188	47.4 (2.9)	49.0	23.0–49.0	0.88
Mother's partner's perceived feelings for the child	269	46.3 (3.4)	48.0	32.0–49.0	0.85	260	46.2 (3.9)	48.0	28.0–49.0	0.88	240	46.6 (4.5)	49.0	15.0–49.0	0.92	188	46.9 (4.0)	49.0	19.0–49.0	0.92
SOC-13	276	71.0 (10.7)	73.5	31.0–89.0	0.86	263	70.1 (12.2)	71.0	27.0–90.0	0.89	239	70.9 (12.5)	73.0	25.0–91.0	0.91	189	70.9 (12.0)	74.0	38.0–90.0	0.91
QDR36	214	25.4 (2.0)	25.7	19.9–29.4	0.90	250	23.8 (2.7)	24.1	13.4–29.2	0.93	229	23.7 (2.9)	24.1	12.6–28.9	0.95	181	23.4 (3.1)	23.7	11.3–29.3	0.95

*Score range*: *Enjoyment of breastfeeding index*: one dimension: range 1–7. *MIRF-scale part one, item “Enjoyment of breastfeeding” included: Mother's perceived relation to the child index*: theoretical range 7–49. *MIRF-scale part one, item “Enjoyment of breastfeeding” not included: Mother's perceived relation to the child index*: theoretical range 7–42. *MIRF-scale part two: Mother's perceived feelings for the child index:* theoretical range 7–49. *Mother's partner's perceived feelings for the child index*: theoretical range 7–49. *SOC-13 index*: theoretical range 13–91; *dimensions*: comprehensibility range 5–35; manageability range 4–28, meaningfulness range 4–28. *QDR36 index:* theoretical range 5–30; *dimensions*: range 1–6. *Values*: *n* = number of participants, *M* = mean; SD = standard deviation; MD = median; *α* = Cronbach's alpha.

**Table 3:** Results from the Mann–Whitney test between first-time mothers who did and did not breastfeed at different points of time throughout the study.

	Q1: breastfeeding during the first two hours after birth	Q1: breastfeeding at the first week after birth	Q2: breastfeeding at six months after birth	Q3: breastfeeding at one year after birth
	*Z* (*p* value), *n* breastfeeding/not breastfeeding	*Z* (*p* value), *n* breastfeeding/not breastfeeding	*Z* (*p* value), *n* breastfeeding/not breastfeeding	Z (*p* value), *n* breastfeeding/not breastfeeding
MIRF-scale part one: Mother's perceived relation to the child index (“Enjoyment of breastfeeding” not included)				
Q1	−0.45 (0.654), 59/171	−1.15 (0.250), 267/13	−0.50 (0.614), 154/92	−1.30 (0.194), 34/190
Q2	−0.20 (0.842), 50/152	−0.41 (0.682), 237/10	−0.62 (0.534), 166/97	−1.14 (0.253), 35/195
MIRF-scale part one: Mother's perceived relation to the child index (“Enjoyment of breastfeeding” included)				
Q1	−0.66 (0.512), 55/169	−0.11 (0.915), 265/5	−0.20 (0.841), 153/84	−1.45 (0.148), 33/183
Q2	−0.43 (0.664), 34/107	−0.62 (0.538), 166/1	−2.49 **(0.013**^*∗*^**)**, 165/13	−2.10 **(0.036**^*∗*^**)**, 34/121
MIRF-scale part two: Mother's perceived feelings for the child index				
Q1	−1.36 (0.174), 56/168	−0.42 (0.674), 260/13	−0.54 (0.589), 149/91	−1.39 (0.164), 33/186
Q2	−0.12 (0.903), 50/152	−1.62 (0.104), 235/10	−0.07 (0.943), 166/95	−2.18 **(0.030**^*∗*^**)**, 34/195
Mother's partner's perceived feelings for the child				
Q1	−0.32 (0.751), 52/165	−0.41 (0.683), 252/13	−0.19 (0.848), 144/90	−1.51 (0.131), 31/184
Q2	−0.66 (0.510), 49/151	1.04 (0.300), 232/10	−0.73 (0.463), 162/96	−1.38 (0.166), 34/194
Breastfeeding or not at				
Q1: first two hours after birth		−3.31 **(0.001**^*∗∗*^**)**, 228/6	−0.34 (0.732), 136/68	
Q1 first week after birth	−3.31 **(0.001**^*∗∗*^**)**, 59/175		−3.58 **(<0.000**^*∗∗*^**)**, 158/91	−1.06 (0.288), 28/158
Q2	−0.34 (0.732), 51/153	−3.58 **(<0.000**^*∗∗*^**)**, 239/10		−0.33 (0.741), 34/193
Q3	−1.06 (0.288), 45/141	−0.33 (0.741), 218/9	−4.26 **(<0.000**^*∗∗*^**)**, 144/87	−4.23 **(<0.000**^*∗∗*^**)**, 35/196
Enjoyment of breastfeeding				
Q1	−1.74 *(0.082#)*, 55/175	−1.76 *(0.079#)*, 271/5	−2.17 **(0.030**^*∗*^**)**, 157/85	−1.60 (0.110), 33/188
Q2	−0.26 (0.791), 34/108	0.00 (1.00), 167/1	−2.78 **(0.005**^*∗∗*^**)**, 166/13	−2.72 **(0.007**^*∗*^**)**, 34/122
Q3	−1.18 (0.239), 14/32	−0.88 (0.376), 55/1	−2.07 **(0.038**^*∗*^**)** 46/11	−1.09 (0.278) 36/24
Willingness to breastfeed a possible following child, Q4	−0.64 (0.523), 45/140	−4.75 **(<0.000**^*∗∗*^**)**, 215/9	−4.72 **(<0.000**^*∗∗*^**)**, 143/85	−2.07 **(0.038**^*∗*^**)**, 36/201
Has had another child two years after birth, Q4	−1.4 (0.154), 35/111	−0.42 (0.672), 177/7	−0.41 (0.686), 130/56	−2.06 **(0.040**^*∗*^**)**, 25/150
SOC-13 index				
Q1	−0.63 (0.532), 55/169	−0.51 (0.609), 261/12	−0.27 (0.791), 151/89	−0.55 (0.586), 33/187
Q2	−0.43 (0.532), 49/152	−0.78 (0.434), 237/9	−0.03 (0.978), 164/98	−0.18 (0.852), 34/195
Q3	−0.78 (0.436), 44/140	−0.40 (0.687), 216/9	−0.30 (0.762), 142/87	−0.25 (0.803), 36/203
Q4	−0.48 (0.634), 35/110	−0.42 (0.676), 176/7	−0.46 (0.649), 130/55	−0.13 (0.894), 25/149
QDR36 index				
Q1	−0.96 (0.338), 41/131	−1.28 (0.201), 203/9	−0.18 (0.855), 119/67	−1.40 (0.163), 25/145
Q2	−0.63 (0.532), 47/149	−0.87 (0.382), 225/9	−0.77 (0.439), 154/95	−0.41 (0.683), 33/186
Q3	−0.48 (0.633), 42/133	−0.87 (0.385), 207/8	−1.22 (0.224), 138/82	−0.88 (0.378), 33/195
Q4	−0.07 (0.945), 33/107	−1.44 (0.149), 169/6	−0.52 (0.603), 123/55	−0.37 (0.710), 23/145

*Questionnaires*: Q1: first week after birth; Q2: six months after birth; Q3: one year after birth; Q4: two years after birth. *Measurements*: MIRF-scale part one, *item “Enjoyment of breastfeeding” included:* Mother's perceived relation to the child index; MIRF-scale part one, *item “Enjoyment of breastfeeding” not included:* Mother's perceived relation to the child index; MIRF-scale part two: Mother's perceived feelings for the child index; and Mother's partner's perceived feelings for the child index. Sense of Coherence (SOC-13); Quality of Dyadic Relationship (QDR36). *Values*: Z: *z*-approximation test. *p* values: ^*∗*^*p* < 0.05; ^*∗∗*^*p* < 0.0, #*p* < 0.1; *n*: number of participants.

**Table 4:** Results from Spearman's correlation analysis for correlated factors with first-time mothers' enjoyment of breastfeeding at six months (Q2) and one year after (Q3) birth, as well as first-time mothers' duration of breastfeeding.

	Enjoyment of breastfeeding at Q2 *r*_*s*_(*p* value), *n*	Enjoyment of breastfeeding at Q3 *r*_*s*_ (*p* value), *n*	Duration of breastfeeding *r*_*s*_(*p* value), *n*
Enjoyment of breastfeeding, Q2	—	0.529 **(0.006**^*∗∗*^**)**, 25	0.241 **(0.005**^*∗∗*^**)**, 133
Enjoyment of breastfeeding, Q3	0.529 **(0.006**^*∗∗*^**)**, 25		0.347 *(0.090#)*, 25
Duration of breastfeeding (weeks)	0.241 **(0.005**^*∗∗*^**)**, 133	0.347 *(0.090#)*, 25	—
Age of participant	−0.146 (0.126), 111	−0.208 (0.424), 17	−0.059 (0.430), 179
Years of education	−0.005 (0.956), 110	−0.264 (0.307), 17	0.043 (0.573), 175
Perceived economy, Q2	0.014 (0.861), 149	0.021 (0.921), 25	0.072 (0.268), 241
Perceived economy, Q3	−0.110 (0.223), 125	−0.092 (0.663), 25	0.045 (0.501), 225
Perceived economy, Q4	−0.020 (0.832), 113	−126 (0.631), 17	0.002 (0.976), 182
Years of parental couple relationship	0.143 (0.191), 85	−0.416 (0.139), 14	0.102 (0.233), 138
MIRF-scale part one: Mother's perceived relation to the child index (“Enjoyment of breastfeeding” included), Q2	−0.048 (0.548), 156	0.361 *(0.076#)*, 25	−0.155 **(0.050**^*∗*^**)**, 159
MIRF-scale part one: Mother's perceived relation to the child index (“Enjoyment of breastfeeding” included), Q3	0.473 **(0.002**^*∗∗*^**)**, 39	0.589 **(0.002**^*∗∗*^**)**, 25	−0.003 (985), 51
MIRF-scale part one: Mother's perceived relation to the child index (“Enjoyment of breastfeeding” included), Q4	0.505 **(0.012**^*∗*^**)**, 24	0.167 (0.789), 5	0.527 **(<0.000**^*∗∗*^**)**, 45
MIRF-scale part one: Mother's perceived relation to the child index (“Enjoyment of breastfeeding” not included), Q2	0.314 **(<0.000**^*∗∗*^**)**, 148	0.445 **(0.026**^*∗*^**)**, 25	−0.006 (0.931), 237
MIRF-scale part one: Mother's perceived relation to the child index (“Enjoyment of breastfeeding” not included), Q3	0.252 **(0.005**^*∗∗*^**)**, 124	0.424 **(0.035**^*∗*^**)**, 25	0.050 (0.460), 224
MIRF-scale part one: Mother's perceived relation to the child index (“Enjoyment of breastfeeding” not included), Q4	0.215 **(0.022**^*∗*^**)**, 113	0.352 (0.165), 17	0.074 (0.318), 182
MIRF-scale part two: Mother's perceived feelings for the child index, Q2	0.359 **(<0.000**^*∗∗*^**)**, 148	0.198 (0.354), 24	0.017 (0.801), 235
MIRF-scale part two: Mother's perceived feelings for the child index, Q3	0.257 **(0.004**^*∗∗*^**)**, 126	0.172 (0.400), 26	0.048 (0.471), 224
MIRF-scale part two: Mother's perceived feelings for the child index, Q4	0.190 **(0.046**^*∗*^**)**, 111	−0.011 (0.968), 16	0.103 (0.171), 179
Mother's partner's perceived feelings for the child index, Q2	0.235 **(0.005**^*∗∗*^**)**, 144	−0.236 (0.267), 24	−0.048 (0.462), 234
Mother's partner's perceived feelings for the child index, Q3	0.115 (0.200), 126	−0.097 (0.639), 26	−0.029 (0.665), 225
Mother's partner's perceived feelings for the child index, Q4	0.138 (0.146) 112	−0.309 (0.227), 17	−0.043 (0.568), 179
SOC-13 index, Q1	0.263 **(<0.000**^*∗∗*^**)**, 264	0.002 (0.978), 130	0.093 (0.178), 213
SOC-13 index, Q2	0.223 **(0.007**^*∗∗*^**)**, 146	0.059 (0.786), 24	0.021 (0.743), 236
SOC-13 index, Q3	0.110 (0.225), 124	0.127 (0.537), 26	−0.012 (0.853), 223
SOC-13 index, Q4	0.122 (0.199), 113	−0.005 (0.986), 17	−0.010 (0.898), 181
QDR36 index, Q1	0.191 **(0.006**^*∗∗*^**)**, 204	−0.043 (0.664), 102	0.240 **(0.002**^*∗∗*^**)**, 164
QDR36 index, Q2	0.097 (0.260), 137	−0.060 (0.780), 24	−0.002 (0.971), 226
QDR36 index, Q3	0.034 (0.712), 119	0.005 (0.793), 25	0.006 (0.932), 214
QDR36 index, Q4	0.069 (0.480), 106	−0.040 (0.884), 16	−0.012 (0.878), 174

*Questionnaires*: Q1: first week after birth; Q2: six months after birth; Q3: one year after birth; Q4: two years after birth. *Measurements*: MIRF-scale part one, *item* “*Enjoyment of breastfeeding” included:* Mother's perceived relation to the child index; MIRF-scale part one, *item “Enjoyment of breastfeeding” not included:* Mother's perceived relation to the child index; MIRF-scale part two: Mother's perceived feelings for the child index; Mother's partner's perceived feelings for the child index; Sense of Coherence (SOC-13); Quality of Dyadic Relationship (QDR36). *Values*: *r*_*s*_: correlation coefficient; *p* values: ^*∗*^*p* < 0.05; ^*∗∗*^*p* < 0.01, #*p* < 0.1; *n ***:** number of participants.

## Data Availability

Data used to support the findings of the study are not available as the research project is still ongoing.

## Supplementary Materials

Supplementary Materials The STROBE guidelines for cohort studies.

## References

[1] Kronborg H., Væth M. (2004). The influence of psychosocial factors on the duration of breastfeeding. *Scandinavian Journal of Public Health*.

[2] Mirkovic K. R., Perrine C. G., Scanlon K. S., Grummer-Strawn L. M. (2014). Maternity leave duration and full-time/part-time work status are associated with US mothers’ ability to meet breastfeeding intentions. *Journal of Human Lactation*.

[3] Dietrich Leurer M., Misskey E. (2015). “Be positive as well as realistic”: a qualitative description analysis of information gaps experienced by breastfeeding mothers. *International Breastfeeding Journal*.

[4] Odom E. C., Li R., Scanlon K. S. (2013). Reasons for earlier than desired cessation of breastfeeding. *Pediatrics*.

[5] World Health Organization (WHO) (2003). *Global Strategy for Infant and Young Child Feeding*.

[6] World Health Organization (WHO) (2019). *Nutrition: Breastfeeding*.

[7] Brockway M., Venturato L. (2016). Breastfeeding beyond infancy: a concept analysis. *Journal of Advanced Nursing*.

[8] Victora C. G., Bahl R., Barros A. J. D. (2016). Breastfeeding in the 21st century: epidemiology, mechanisms, and lifelong effect. *The Lancet*.

[9] The National Board of Health and Welfare (Sweden) (2017). *Statistics on Breastfeeding 2016. Health and Medical Care*.

[10] Teich A. S., Barnett J., Bonuck K. (2013). Women’s perceptions of breastfeeding barriers in early postpartum period: a qualitative analysis nested in two randomized controlled trials. *Breastfeeding Medicine*.

[11] Palmér L., Carlsson G., Mollberg M. (2010). Breastfeeding: an existential challenge women’s lived experiences of initiating breastfeeding within the context of early home discharge in Sweden. *International Journal of Qualitative Studies on Health and Well-Being*.

[12] Palmér L., Carlsson G., Mollberg M. (2012). Severe breastfeeding difficulties: existential lostness as a mother—women’s lived experiences of initiating breastfeeding under severe difficulties. *International Journal of Qualitative Studies on Health and Well-Being*.

[13] Krol K. M., Grossmann T. (2018). Psychological effects of breastfeeding on children and mothers. *Bundesgesundheitsblatt—Gesundheitsforschung—Gesundheitsschutz*.

[14] Antonovsky A. (1993). The structure and properties of the sense of coherence scale. *Social Science & Medicine*.

[15] Ngai F.-W., Ngu S.-F. (2014). Family sense of coherence and family adaptation among childbearing couples. *Journal of Nursing Scholarship*.

[16] Ngai F.-W., Ngu S.-F. (2015). Predictors of maternal and paternal depressive symptoms at postpartum. *Journal of Psychosomatic Research*.

[17] Ngai F.-W., Ngu S.-F. (2016). Family sense of coherence and family and marital functioning across the perinatal period. *Sexual & Reproductive Healthcare*.

[18] Cortelo F. M., Marba S. T. M., Cortellazzi K. L. (2018). Women’s sense of coherence and its association with early weaning. *Jornal de Pediatria*.

[19] Antonovsky A. (1987). *Unraveling the Mystery of Health*.

[20] Volanen S.-M., Suominen S., Lahelma E., Koskenvuo M., Silventoinen K. (2007). Negative life events and stability of sense of coherence: a five-year follow-up study of Finnish women and men. *Scandinavian Journal of Psychology*.

[21] Ahlborg T., Berg S., Lindvig J. (2013). Sense of coherence in first-time parents: a longitudinal study. *Scandinavian Journal of Public Health*.

[22] Hildingsson I. (2017). Sense of coherence in pregnant and new mothers—a longitudinal study of a national cohort of Swedish speaking women. *Sexual & Reproductive Healthcare*.

[23] Bäckström C., Kåreholt I., Thorstensson S., Golsäter M., Mårtensson L. B. (2018). Quality of couple relationship among first-time mothers and partners, during pregnancy and the first six months of parenthood. *Sexual & Reproductive Healthcare*.

[24] Bäckström C. A., Mårtensson L. B., Golsäter M. H. (2016). “It’s like a puzzle”: pregnant women’s perceptions of professional support in midwifery care. *Women Birth*.

[25] Bäckström C., Thorstensson S., Mårtensson L. B. (2017). To be able to support her, I must feel calm and safe’: pregnant women’s partners’ perceptions of professional support during pregnancy. *BMC Pregnancy Childbirth*.

[26] Bäckström C., Larsson T., Wahlgren E., Golsäter M., Mårtensson L. B., Thorstensson S. (2017). It makes you feel like you are not alone’: expectant first-time mothers’ experiences of social support within the social network, when preparing for childbirth and parenting. *Sexual & Reproductive Healthcare*.

[27] Bäckström C. A. (2018). Professional and social support for first-time mothers and partners during childbearing.

[28] Thorstensson S., Hertfelt Wahn E., Ekström A. (2012). Evaluation of the mother-to-infant relation and feeling scale: interviews with first-time mothers for feelings and relation to their baby three days after birth. *International Journal of Nursing and Midwifery*.

[29] Ekström-Bergström A., Nissen E. A. (2006). A mother’s feelings for her infant are strengthened by excellent breastfeeding counseling and continuity of care. *Pediatrics*.

[30] Thorstensson A., Claesson A., Packalen A. (2014). Validating the mother-to-infant relation and feelings’ scale by first-time mothers’ descriptions three months after birth. *Journal of Women’s Health, Issues & Care*.

[31] Antonovsky A. (1996). The salutogenic model as a theory to guide health promotion. *Health Promotion International*.

[32] Langius A., Björvell H., Antonovsky A. (1992). The sense of coherence concept and its relation to personality traits in Swedish samples. *Scandinavian Journal of Caring Sciences*.

[33] Ferguson S., Browne J., Taylor J., Davis D. (2016). Sense of coherence and women׳s birthing outcomes: a longitudinal survey. *Midwifery*.

[34] Ekström A., Widström A.-M., Nissen E. (2003). Duration of breastfeeding in Swedish primiparous and multiparous women. *Journal of Human Lactation*.

[35] Ahlborg T., Lilleengen A.-M., Lönnfjord V., Petersen C. (2009). Quality of dyadic relationship in Swedish men and women living in long-term relationships and in couples in family counselling - introduction of a new self-report measure, QDR36. *Nordic Psychology*.

[36] Ahlborg T., Persson L.-O., Hallberg L. R.-M. (2005). Assessing the quality of the dyadic relationship in first-time parents: development of a new instrument. *Journal of Family Nursing*.

[37] Figueiredo B., Canário C., Field T. (2014). Breastfeeding is negatively affected by prenatal depression and reduces postpartum depression. *Psychological Medicine*.

[38] Watkins S., Meltzer-Brody S., Zolnoun D., Stuebe A. (2011). Early breastfeeding experiences and postpartum depression. *Obstetrics & Gynecology*.

[39] Emerson J. A., Tol W., Caulfield L. E., Doocy S. (2017). Maternal psychological distress and perceived impact on child feeding practices in South Kivu, DR Congo. *Food and Nutrition Bulletin*.

[40] Kendall-Tacket K. A. (2016). *Depression in New Mothers. Causes, Consequences and Treatment Alternatives*.

[41] Webb H. J. (2018). Maternal self-rated health and psychological distress predict early feeding difficulties: results from the longitudinal study of Australian children. *International Journal of Eating Disorders*.

[42] Cassidy J., Cassidy J., Shaver P. R. (1999). The nature of the child’s ties. *Handbook of Attachment: Theory, Research and Clinical Applications*.

[43] Fowles E. R., Horowitz J. A. (2006). Clinical assessment of mothering during infancy. *Journal of Obstetric, Gynecologic & Neonatal Nursing*.

[44] Mercer R. T., Walker L. O. (2006). A review of nursing interventions to foster becoming a mother. *Journal of Obstetric, Gynecologic & Neonatal Nursing*.

[45] Bigelow A. E., MacLean K., Proctor J., Myatt T., Gillis R., Power M. (2010). Maternal sensitivity throughout infancy: continuity and relation to attachment security. *Infant Behavior and Development*.

[46] Spanier G. B. (1976). Measuring dyadic adjustment: new scales for assessing the quality of marriage and similar dyads. *Journal of Marriage and Family*.

[47] De Lauzon-Guillain B., Wijndaele K., Clark M. (2012). Breastfeeding and infant temperament at age three months. *PLoS One*.

[48] Al-Sahab B., Lanes A., Feldman M. (2010). Prevalence and predictors of 6-month exclusive breastfeeding among Canadian women: a national survey. *BMC Pediatrics*.

[49] Kronborg H., Foverskov E., Vaeth M. (2018). The role of intention and self-efficacy on the association between breastfeeding of first and second child, a Danish cohort study. *BMC Pregnancy and Childbirth, England*.

[50] Magnusson M., Lagerberg D., Wallby T. (2016). No widening socioeconomic gap within a general decline in Swedish breastfeeding. *Child: Care, Health and Development*.

[51] Colodro-Conde L., Sánchez-Romera J. F., Tornero-Gómez M. J., Pérez-Riquelme F., Polo-Tomás M., Ordoñana J. R. (2011). Relationship between level of education and breastfeeding duration depends on social context. *Journal of Human Lactation*.

[52] Scott J. A., Shaker I., Reid M. (2004). Parental attitudes toward breastfeeding: their association with feeding outcome at hospital discharge. *Birth*.

[53] Scott J. A., Kwok Y. Y., Synnott K. (2015). A comparison of maternal attitudes to breastfeeding in public and the association with breastfeeding duration in four European countries: results of a cohort study. *Birth*.

[54] Laanterä S., Ekström A., Pölkki T. (2010). Breastfeeding attitudes of Finnish parents during pregnancy. *BMC Pregnancy and Childbirth*.

[55] Bessette-Symons B. A. (2018). The robustness of false memory for emotional pictures. *Memory*.

